# Evaluation of Neglected Idiopathic Ctev Managed by Ligamentotaxis Using Jess: A Long-Term Followup

**DOI:** 10.4061/2011/218489

**Published:** 2010-10-19

**Authors:** Ajai Singh

**Affiliations:** Department of Orthopaedics, C. S. M. Medical University, 2/59, Viram Khand, Gomti Nagar, 226010 Lucknow, India

## Abstract

*Background*. This study was conducted with the aim of evaluating the role of Ligamentotaxis in the management of neglected clubfeet managed by ligamentotaxis using Joshi's External Stabilisation System (JESS). *Method & Material*. Total 20 subjects (28 feet) were studied, which were corrected by differential ligamentotaxis using JESS. All were evaluated clinically, radiologically, podogrammically, and by Catterall Pirani Scoring System, both before and after the correction. *Results*. Severity of the deformities and clinical correction was assessed by Pirani score. All patients achieved good clinical results as per Pirani score, which was statistically significant. Radiological evaluation showed that all subjects achieved the normal range of values. The pre- and postcorrection difference in FBA was statistically significant. *Conclusion*. Differential distraction by fixator for the correction of neglected idiopathic CTEV is an effective and patient-friendly method of management.

## 1. Introduction

The CTEV, a hereditary foot deformity is one of the commonest congenital foot anomalies presenting to a paediatric orthopaedic surgeon. Its incidence is 5–6 per 1000 live births, varying with race and geography [1]. The goal of any type of CTEV management is to reduce, if not to eliminate all elements of the clubfoot deformity, hence achieving a functional, pain free, normal looking plantigrade, mobile, callous free, and normally shoeable foot [1]. The various factors that have been associated with the poor prognosis in CTEV management are female child, hereditary, late age of presentation, severity of deformity, rigidity of foot, associated cavus, associated clawing of toes, and small heel [2–6]. Kite [7] rationalized the whole treatment of clubfoot by conservative means. Recently, various workers have shown satisfactory results by Ponseti [8] method of manipulation and serial casting. With the fear of possible complications of open surgery, minimally invasive surgery had been advocated long back for correcting the clubfoot deformity. Percutaneous soft tissue release and tenotomy for getting the corrected foot had been advocated by various workers [5]. The method of controlled differential distraction, that is, ligamentotaxis, along with the miniexternal fixator was originally described by Dr. B. B. Joshi in 1990. Ilizarov fixator [2] has also been used for correction of CTEV deformities. Recently associations of internal talar spin and varus component of this deformity has been established [6]. Clinically the talar spin can be measured by foot bimalleolar axis [6]. We considered any clubfoot presented first time to us for the management at or after the age of 01 year. Although “neglected” cases have not been defined in the literature, we considered any patient presenting to us after the age of 03 years as late presentation/neglected cases. This study was conducted to evaluate the clinico-radiological outcomes of neglected idiopathic CTEV managed by ligamentotaxis using JESS.

## 2. Material and Method

This observational study was conducted on all the patients with late presentation of CTEV since July 2003 to January 2005. All patients of 03–06 years of age of both sexes with idiopathic CTEV feet fulfilling following criteria, such as presenting first time for the management of clubfoot in our OPD, patients managed earlier but not fully corrected, and all previous conservatively corrected clubfoot presented with relapse of deformity, were included. We excluded patients below 03 years and above 06 years of age and if associated with secondary causes like arthrogryposis, meningomyelocele, and so forth. All included patients were assessed and managed by author only. All patients included in this study were thoroughly assessed clinically including podograms and radiologically. In the radiological assessments, measurements of various angles were done in AP and lateral view in stress dorsiflexion in all cases. X rays were studied for talocalcaneal angle, talo-first metatarsal angle, talo-Vth metatarsal angle (all in AP view), talocalcaneal angle, Tibiocalcaneal angle and Calcaneal pitch (all in lateral view). Catterall Pirani scoring system was used in this study to assess the severity of deformity and to assess the correction achieved after final casting. Podograms were taken to assess the weight bearing portion of foot, length, and width of foot before and after completion of treatment. After keeping the foot in weight bearing position, the foot tracings were taken on a plain white paper. Simultaneously the midpoints of both malleoli were marked on the same footprint by placing a pencil on both sides. A long “axis of foot” was drawn taking 2nd toe and midpoint of most broad part of heel as the two reference points. A line joined the two medial malleoli marks known as “bimalleolar axis”, which intersect with this long axis of foot. Anteromedial angle of the intersection was taken as “Foot Bimalleolar Angle” (FBA). As described in the literature, the normal value of FBA is 82.5°. Feet were classified in groups I, II, III as per the Jain et al. study [23] (group I: >73.2°, group II: >  66.6–73.2° and group III: <66.6°). FBA was recorded before and after the treatment. After this assessment, all these feet were manipulated by Ponseti technique. Those feet showed significant clinical improvement after 04 manipulations, were excluded from the study and rest feet, not responding to the manipulations, were then included in the study, and were operated (JESS–external fixator assembly).

**Figure 1:**
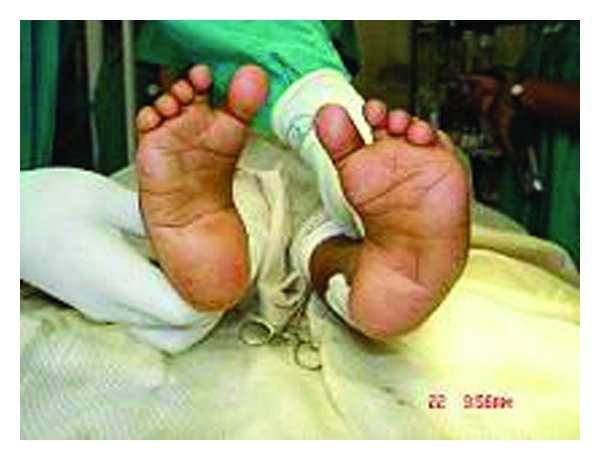
Clinical photo showing deformities in both feet.

**Figure 2:**
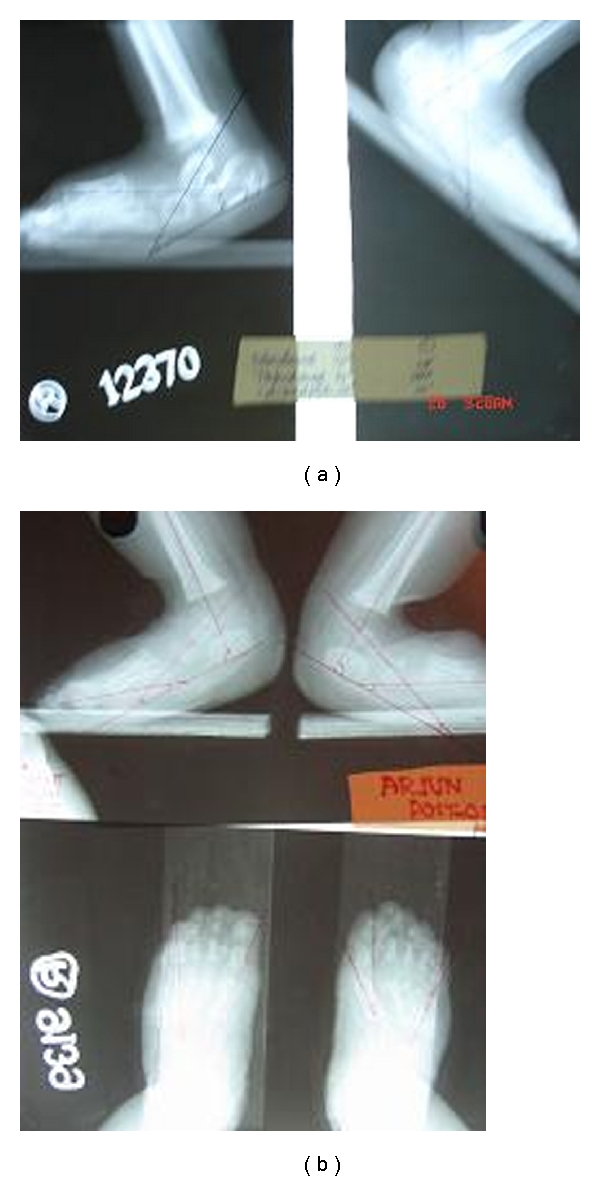
(a) X-rays AP and Lateral (stress dorsiflexion) of feet showing extend of various abnormal radiographic angles preoperatively. (b) X-rays AP and Lateral (stress dorsiflexion) of feet showing extend of various abnormal radiographic angles postoperatively.

**Figure 3:**
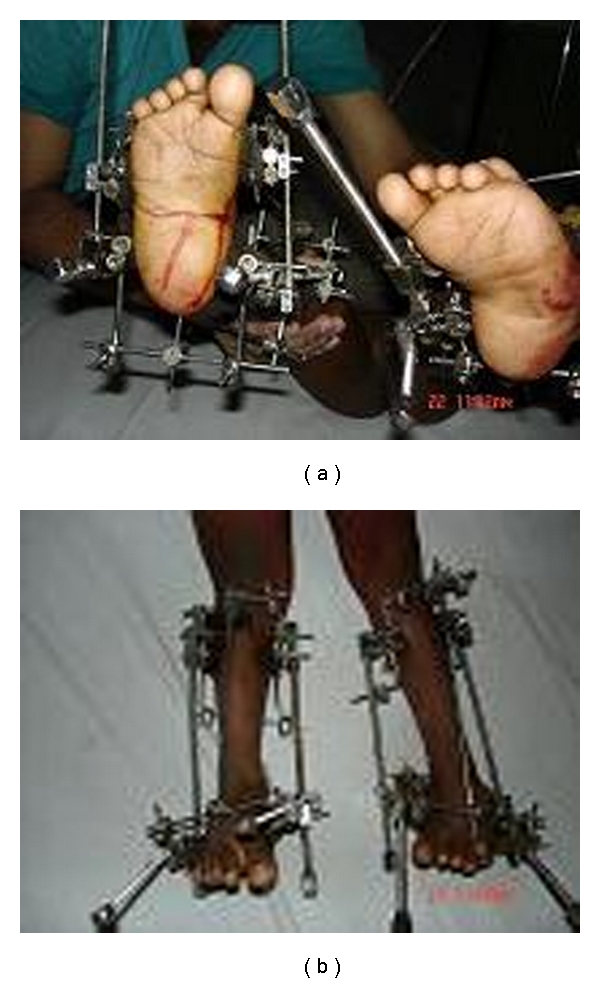
Clinical photos showing the fixator assembly in position before ligamentotaxis is started.

**Figure 4:**
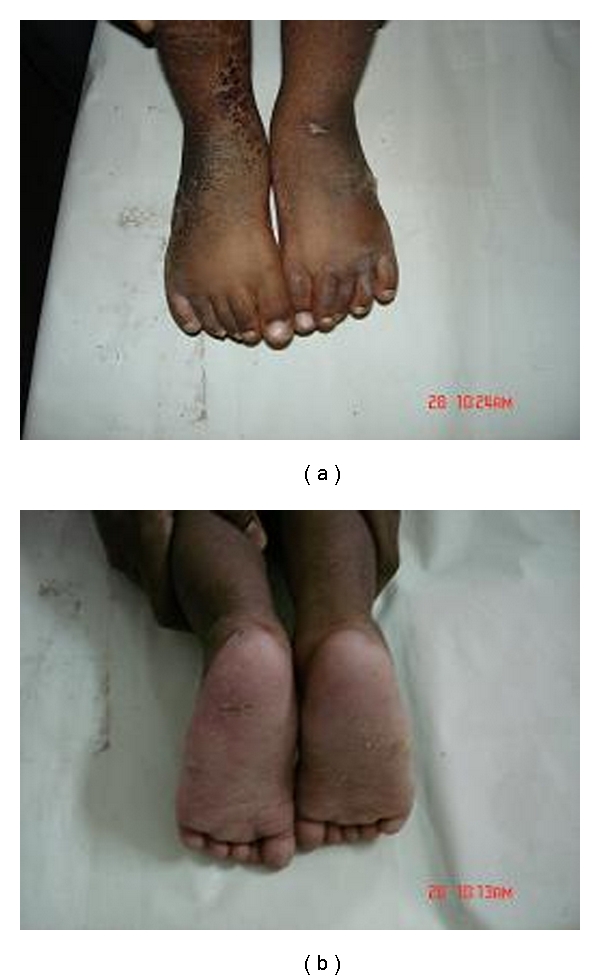
Clinical photos showing correction of deformities achieved by ligamentotaxis.

**Figure 5:**
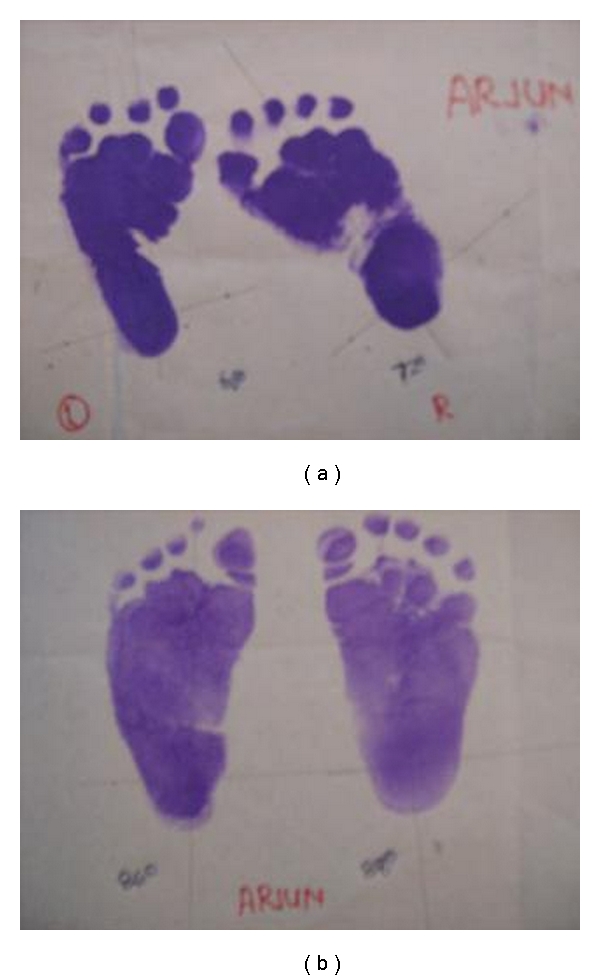
(a) Precorrection podograms showing reduced foot bimalleolar angles. (b) Postcorrection podograms showing improved foot bimalleolar angles achieved by ligamentotaxis.

 We operated all our patients in general anaesthesia. After putting all the K-wires (i.e., 3 each tibial, calcaneal, and metatarsal), we tried to correct the deformity by standard Ponseti method and then maintained whatever reduction we achieve, by completing the frame by connecting the tibial, calcaneal, and metatarsal attachments. One each distractor was placed on both side between tibial-calcaneal and calcaneal-metatarsal attachments. The cavus, if any, was corrected by subcutaneous tenotomy. The distraction was started on the third postoperative day in standard manner, that is, 0.25 mm four times a day on the medial side while 0.25 mm two times a day on the lateral side. We continued this gradual fractional distraction till we achieve the clinical overcorrection (Figure 1, 2, 3, 4, and 5). At this point we removed the fixator and again radiological assessment was done. Podograms were also obtained (and FBA was measured). Then the measurements for corrected shoes and/or D B splint were taken and AKPOP in overcorrected position was given for next 02 weeks. After the 02 weeks we gave orthosis and/or splint and patients were followed up regularly. The importance of bracing was emphasized to the parents and they were advised to comply strictly with the bracing protocol. At the end of 06 months, 12 months, 18 months, and 24 months, all clinical assessments were done and documented. Radiological assessment was also done at the end of 01 year followup and was analysed. After 24 month followup patients were told to contact for followup annually. They were told to report in case of relapse of any deformity. Cases were considered as failure if (a) there was no or incomplete clinico-radiological correction or (b) complications like joint subluxation, rocker bottom deformity occurred.

## 3. Observations and Results

We managed 33 neglected idiopathic CTEV feet were by Ponseti technique, out which only 5 (15.1%) feet were responded to these manipulations. Rest total 28 feet in 20 patients were included in the study. There were 14 male and 6 female patients. The minimum age was 3.4 years and maximum was 5.2 years (mean age −  4.2 years). All 28 feet had severe clinical deformities (clinical grade III, Pirani score 5-6 and FBM angle below 66 degrees). Total 22 feet were managed previously elsewhere by corrective manipulations with plaster, 02 were operated elsewhere (posteromedial soft tissue release) and rest were never received any mode of treatment. Mean precorrection FBA (60.9 degrees) was corrected to 78.7 deg. Mean preoperative TC index (19.2), improved to 63.1. All other clinico-radiological parameters were also improved (statistical significant) in all patients. Only 6 (18.7%) feet developed superficial infection (not severe enough compelling any active intervention). Only 10 (31.2%) feet presented with relapsed forefoot adduction (corrected by manipulations and retention by plasters in all cases) and all returned to orthosis. No open correction of any component of deformity in any case at any stage was done. 

 The mean precorrection equinus deformity was 57°. The mean dorsiflexion achieved after correction was 16.4° in these patients. The mean precorrection adduction deformity was 28° and the mean postcorrection abduction achieved was 3° in these patients. The mean precorrection heel varus was 41.3° while the mean postcorrection value of varus was 4.5°. Before correction 05 (17.9%) feet had cavus deformity, which was corrected in all of these patients. The mean precorrection Sinha index was 0.7 and after correction the mean Sinha index achieved was 1.07. In unilateral cases, average difference in the calf size of affected side and normal side was 1.0 cm while the calf size was same in bilateral cases. The calf size remain unaffected by the procedure. 

 To evaluate our end results, the subjects were graded on a scale of good to poor using Pirani Score. A final Pirani score of 0–2 is regarded as good clinical correction achieved. All patients were reverted to 0–2 group, that is, good outcome. Before correction the mean Pirani Total score was 5, which was reduced to 0.7 after the correction, that is, all became more flexible than earlier. By the end of the followup the flexibility of the feet remained unchanged.

## 4. Discussion

Congenital Talipes Equinovarus is a common paediatric orthopaedic problem, which constitutes a bulk of the congenital anomalies presenting to any paediatric orthopaedic surgeon. 

 Various methods of management [9–22] of these feet, including conservative treatment (Ponseti technique of manipulation with plaster) have been reported in the literature, with variable success rates. Many papers are published now successfully using ponseti method alone to correct neglected clubfeet as well as clubfeet that have had previous extensive surgery and then relapsed. Surgery had been mainly advocated for late, neglected, and relapsed feet [2], but many workers had shown advantages of minimal to extensive surgery in early cases also.

 The Ponseti technique [3, 6, 9] had been accepted by many orthopaedic surgeons as method of choice to manipulate and correct these feet. They were of the opinion that the early correction can be achieved in these feet with a low recurrence rate. The explanation given for better deformity correction by Ponseti technique is that (a) pronation should never be done as it causes the calcaneum to jam under talus. The calcaneum does not rotate and remains in varus, (b) by using Ponseti technique, calcaneum is allowed to rotate under the talus, which also is free to rotate in ankle mortise. This is achieved by abducting the forefoot in supination with the counter pressure on lateral aspect of head of talus [6]. The philosophy of this technique is that the center of CTEV deformity lies with head of talus with a medial talar spin, which can be measured by FBA. Khan and Kumar [9] evaluated the efficacy of Ponseti's technique in 25 neglected clubfoot in children more than 07 years of age (mean age 8.9 years). The mean followup period was 4.7 years. The observed 85.7% of feet were fully corrected with recurrence in 24% of feet. 

 But several surgeons are now discarding this method along with other soft tissue surgeries in favour of distraction as differential distraction had shown a distinct and rare advantage that in addition to deformity correction; it also produces a cosmetic foot with near normal foot size. With the same concept, we too managed these feet by differential distraction by JESS. As it does not require any open or percutaneous surgical procedure for the deformity correction, it has been labeled as “extended conservative management”. After the desired clinical correction achieved, foots were supported in maximum corrected position in AKPOP cast for next 04 weeks and then were put on DB splint. the only major drawback we felt was the acceptance assembly by the children. Another drawback was the chances of injuries to the children and their attendants while nursing. On the overall assessment the results are quite encouraging yielding good correction in much short period. We also observed that correction continued even after the fixator removal. Although we do not have any explanation for it, our hypothesis was that the postdistraction neo-osteogenesis occurs which somewhat resemble the normal tissue. 

 Our controlled differential distraction assembly differs from the classical Ilizarov technique in significant aspects. (1) Axially tensioned wires are not used in our frame. (2) Clubfoot is a multiplanar, multiapical deformity. It is very difficult to plan the location of an external hinge for deformity correction. Our frame is unconstrained and relies on correction occurring at the natural joints. (3) Differential distraction is used to correct the deformity. This achieves deformity correction without compressing the child's foot. 

 We excluded patients below the age of 03 year with the fear that their soft bones may not be able to bear the distraction forces. We were of the opinion that the children below this age could be treated by lesser extensive approach (Ponseti). We also excluded patients above 06 years as by this age, the significant bony changes may affect the outcome. 

 In present study, improvement in Medial/Lateral border ratio was observed in all subjects although we were not able to achieve complete reversal of medial to lateral border ratio, as probably the duration of observation was short. In unilateral cases, affected foot though remains smaller in comparison to the normal foot but was cosmetically acceptable to all parents. 

 As far as analysis of FBA parameter is concerned, Jain et al. [23] showed improvement from grade III to grade I in 93% of cases managed by Ponseti manipulation in early cases, while in present study we were able to bring FBA grade III to FBA grade I in 87.1% of our cases.

 As per our observations, radiological parameters return to normal range. The possible explanation for this could be that the primary pathology in CTEV is soft tissue contractures around midfoot and hindfoot while the bony articulation changes are not initially marked as skeleton is mainly cartilaginous. The purpose of distraction is to stretch the contracted ligaments gradually and differentially. The difference in pre- and postcorrection Pirani scores in these patients was found statistically significant (*P* = 0.01).

 Thirty four cases of severe relapsed and neglected clubfoot deformity were treated with Ilizarov fixator [24]. Good results were achieved in about 58.8% of cases with recurrence in about 8.7%. A study on 44 neglected clubfoot [25] managed by JESS distractor and followed up for minimum period of 2 years, had obtained about 90% excellent to good. In a study [26], 41 children with idiopathic neglected CTEV, residual CTEV or recurrent CTEV were managed by JESS and followup for 3.6 years. They obtained 59.7% excellent and good results.

 Though it is a very small series but by far we are able to achieve very encouraging and comparable results. We may conclude that correction of late presented CTEV by ligamentotaxis is patients and surgeon friendly procedure. But in this procedure the active participation of the patients' attendants is one of the prime factors for the successful outcome.
